# The Modifying Effect of Individual-Level Neighborhood Perceptions on the Relationship Between Census-Level Neighborhood Social Vulnerability and Cognition in Older Latinos

**DOI:** 10.3390/ijerph23060714

**Published:** 2026-05-27

**Authors:** Melissa Lamar, Cheyenne V. Parson, Crystal M. Glover, Ana W. Capuano, Mayra L. Estrella, Emily P. Morris, Lisa L. Barnes, David A. Bennett, David X. Marquez

**Affiliations:** 1 Rush Alzheimer’s Disease Center, Rush University Medical Center, Chicago, IL 60612, USA; mayra_estrella@rush.edu (M.L.E.); emily_morris@rush.edu (E.P.M.); lisa_l_barnes@rush.edu (L.L.B.); david_a_bennett@rush.edu (D.A.B.); marquezd@uic.edu (D.X.M.); 2 Department of Psychiatry and Behavioral Sciences, Rush University Medical Center, Chicago, IL 60612, USA; 3 School of Public Health, University of Illinois Chicago, Chicago, IL 60612, USA; cpars@uic.edu; 4 Department of Neurology, University of California, Irvine, CA 92697, USA; glover1@hs.uci.edu; 5 Department of Neurology, The University of Chicago, Chicago, IL 60637, USA; capuano@uchicago.edu; 6 Department of Internal Medicine, Rush University Medical Center, Chicago, IL 60612, USA; 7 Department of Neurological Sciences, Rush University Medical Center, Chicago, IL 60612, USA; 8 Department of Kinesiology and Nutrition, University of Illinois Chicago, Chicago, IL 60612, USA

**Keywords:** neighborhood health, social vulnerability, cognition, perceptions, Latinos

## Abstract

**Highlights:**

**Public health relevance—How does this work relate to a public health issue?**

- Increasingly, some—but not all—studies suggest residents of highly disadvantaged or vulnerable neighborhoods show lower levels of cognition making neighborhood health a key focus of public health.

**Public health significance—Why is this work of significance to public health?**

- By investigating discrepant associations of Census-level measures with cognition, including our own null results, within the context of individual-level neighborhood perceptions, we may provide alternative targets for public health interventions.

**Public health implications—What are the key implications or messages for practitioners, policy makers and/or researchers in public health?**

- Results demonstrate the importance of incorporating individual-level neighborhood perceptions when researching Census-level measures of neighborhood health and cognition and support implementing improvement projects in vulnerable areas that focus not only on rebuilding physical infrastructure but also on creating engaging environments.

**Abstract:**

Residents of vulnerable neighborhoods show lower levels of, and faster longitudinal declines in cognition across most, but not all studies. Mixed results may exist, in part, because residents’ self-reported (individual-level) perceptions of their neighborhood do not always reflect Census-level measurements. We investigated both types of neighborhood characteristics to reexamine previously reported null associations between neighborhood vulnerability and cognition within an interactive socio-environmental framework. Self-identified Latinos (*N* = 224; $\bar{x}$age = 70.4) without dementia completed a modified Perception of Neighborhood Environment Scale (mPNES; higher scores = more positive perceptions of total and domain-specific community cohesiveness, health opportunities, and ambience), annual cognitive assessments, and had baseline addresses geocoded to a Census-derived Social Vulnerability Index (SVI; higher scores = higher vulnerability). Separate mixed effects regression models adjusted for relevant confounders tested relationships between mPNES and cognitive composite z-scores, and modifying effects of mPNES on the SVI-cognition associations. Higher total mPNES scores were associated with higher baseline global cognition and working memory (driven by community cohesiveness). Higher domain-specific health opportunities associated with slower rates of global cognitive decline. mPNES modified relationships between SVI and cognition, particularly baseline global cognition and episodic memory. Results demonstrate the importance of incorporating individual-level neighborhood perceptions when examining relationships between Census-level measures of neighborhood health and cognition.

## 1. Introduction

Research incorporating the exposome, i.e., physical, chemical, biological, and (psycho)social influences in the environment [1], into studies of cognitive aging and dementia has significantly increased in the past decade. This is, in part, thanks to freely available data on exposures including neighborhood deprivation (e.g., Area Deprivation Index; [2,3]) and vulnerability (e.g., Centers for Disease Control’s Social Vulnerability Index, CDC SVI; [4]). Residents of highly disadvantaged or vulnerable neighborhoods often show lower levels of cognition [3,5,6], faster rates of cognitive decline [7,8], and higher risk of Alzheimer’s disease (AD) and AD-related dementias (ADRD) [8,9]. Some studies [10], including our own [6], have not reported these deleterious associations for all high-risk groups. For example, older Latinos participating in our cohort studies did not show associations between Census-derived neighborhood vulnerability and cognitive functioning [6]; this was despite living in more vulnerable areas compared to same-aged peers who did show these associations [5,6,11].

One potential explanation for these discrepant findings is that Census-level measures of neighborhood health do not always match resident’s individual-level, i.e., self-reported perceptions of these same indicators (see [12] for review). Furthermore, when considered together, individual-level neighborhood measures do not always show similar associations with adverse outcomes as Census-level measures (e.g., [13]). Thus, while Census-level measures of neighborhood health may intimate one type of environment, residents’ perceptions of these same characteristics often show little agreement [12]. Furthermore, recent studies of both aspects of neighborhood health report that negative individual-level perceptions may confer an increased risk of adverse outcomes—including cognitive outcomes—beyond Census-level neighborhood disadvantage [14,15]. Given that perceptions of neighborhood health are associated with cognitive health across several diverse populations including Latinos [16,17], and work is emerging to suggest that these individual-level perceptions are associated with cognition in ways Census-level measures are not (e.g., [18]), more work is needed incorporating both aspects of neighborhood health within the same study.

A more comprehensive incorporation of neighborhood factors across levels of analysis is in keeping with several enduring socio-environmental models of health and aging [19,20]. These models advocate for a more layered consideration of the multiple interconnected pathways that shape adverse outcomes including cognition [19,20], particularly in high-risk communities [21,22]. Thus, resident-based individual-level perceptions of neighborhood health capture unique experiential dimensions of environmental and social conditions, providing complimentary and even clarifying information to Census-level studies of neighborhood health [15,23,24]. Considering resident’s individual-level neighborhood perceptions may be particularly important among Latinos. First, the previously described null findings for Census-level neighborhood health and cognition [6] need exploration. Second, there are mixed findings on whether neighborhood factors are stronger or weaker in primarily Latino neighborhoods (e.g., social cohesion) [25]. Third, unique cultural factors (e.g., the importance of family and the protective effect of ethnic enclaves) may contribute to individual-level neighborhood perceptions for Latinos. This study is the first step toward addressing these considerations.

In the current study, we investigated individual- and Census-level neighborhood health measures to reexamine previously reported [6] null associations between a Census-derived neighborhood Social Vulnerability Index (SVI) and cognition in older Latinos. Specifically, we first investigated associations between a modified version of the self-report Perception of Neighborhood Environment Scale [26] and baseline levels of as well as longitudinal changes in global and domain-specific cognition. We then tested the modifying effect of neighborhood perceptions on associations between the SVI and these same cognitive outcomes. Given prior null results between SVI and cognition [6], we felt it more appropriate to investigate effect modification instead of mediation. Not only is this decision consistent with recent recommendations in the literature [27], it is in line with socio-environmental theories suggesting these levels of neighborhood health are equally important and interacting [19,20]. Thus, we hypothesize that participants rating their neighborhoods more positively will have higher baseline cognition and a slower rate of cognitive decline (most apparent for domains of learning and memory as suggested by prior research, e.g., [16,17,28]). We further hypothesize that individual-level perceptions of neighborhood health will modify associations between Census-level measures of neighborhood health and cognition given their role in other studies [16,17,25,28,29], unmasking previously unreported associations and modifying others.

## 2. Materials and Methods

### 2.1. Participants

Participants were self-identified older Latinos enrolled in one of four community-based cohort studies at the Rush Alzheimer’s Disease Center (RADC). Specifically, these studies included the Latino Core (2016 to present) [30], the Clinical Core (2004 to present) [31], the Rush Memory and Aging Project (MAP, 1997 to present) [32], and the Minority Aging Research Study (MARS; 2004 to present) [33]. All studies are ongoing longitudinal studies of cognitive aging that recruit from community-based settings specifically catered to older adults in the Chicagoland region. Participants agree to annual in-home evaluations that include harmonized questionnaires and cognitive testing as outlined below. All procedures are administered by the same study staff to ensure data may be merged across studies. An Institutional Review Board of Rush University Medical Center approved all cohort studies and participants provided written informed consent following the Declaration of Helsinki.

Of the participants who self-identified as Latino from the named cohort studies above, 430 completed study baseline and 306 completed our subjective individual-level measure of neighborhood health first implemented in 2020. While 285 of these 306 participants had a valid geocoded address at baseline, 42 participants lacked objective Census-level neighborhood health scores given that the visit year coinciding with their baseline address fell outside of the years of available data. This left 243 older Latinos eligible for this project. We excluded 19 participants with dementia at the time they completed their self-reported subjective neighborhood health questionnaire. Dementia was based on a uniform structured clinical evaluation [34] and National Institute of Neurological and Communicative Disorders and Stroke and the Alzheimer’s Disease and Related Disorders Association dementia criteria [35]. The 224 remaining participants all had at least two study visits to facilitate longitudinal analyses. More specifically, participants had a mean of 6.77 (SD = 2.47; max = 17) study visits from geocoded (i.e., analytic) baseline.

### 2.2. Subjective Neighborhood Health

A shortened version of the original Perceived Neighborhood Environment Scale [26] was used to measure individual-level experiences of neighborhood health. This scale was originally developed to understand how perceptions of neighborhood-level dimensions including esthetic quality, walking environment, food availability, social cohesion, and safety relate to cardiovascular health. We shortened this 36-item scale to reduce participant burden while also maintaining the comprehensive coverage of neighborhood dimensions. Specifically, decisions were made on items to retain based on the text of the original article [26], a directed review of the larger literature, and consultation with cohort study teams. We were careful to select and maintain key indicators representing each of the categories outlined in the original PNES where possible. We statistically investigated the alignment of our choices to the original questionnaire using pilot data from a small group of older adults (*N* = 81; 58% non-White) who completed the 36-item scale and to data outlined in the original article [26]. From this emerged a 12-item modified Perceived Neighborhood Environment Scale (mPNES) using the same Likert-scale from 1 (Strongly Agree) to 5 (Strongly Disagree) to rate a neighborhood similarly defined as “the area within about a 20 min walk (or about a mile) from your house” as the original. Responses were reverse-coded as needed and averaged to create a total score with higher scores (max score = 5) reflecting more positive perceptions of neighborhood health.

Given our desire to maintain as much fidelity to the original scale [26] as possible, in addition to the total score we also created domain-specific mPNES scores. As outlined in more detail elsewhere [36], a principal component analysis (PCA) with varimax rotation determined appropriate subscales for all RADC participants using their first valid time point of mPNES data. The PCA-derived factor loadings with eigenvalues greater than 1.0 reflected (1) community cohesiveness, (2) health opportunities, and (3) ambient environments and accounted for 63% of the total variance. These loadings were used to create the domain-specific subscales. Specifically, the six items of the community cohesiveness sub-scale reflected three questions pertaining to perceptions of social cohesion and a person’s relationship with their neighbors (e.g., “People in my neighborhood generally get along with each other”) and three questions pertaining to the esthetic quality of the neighborhood more generally (e.g., statements about neighborhood attractiveness, order, and walkability). The health opportunities sub-scale totaled four questions and queried perceived availability of healthy food (e.g., “A large selection of fresh fruits and vegetables is available in my neighborhood”), fitness facilities, and other “opportunities to be physically active”. Lastly, perceptions of the ambient environment focused on two questions related to traffic and noise (e.g., “There is a lot of noise in my neighborhood”). Item-level data was averaged, as relevant, for domain-specific mPNES scores (max score = 5; higher score = higher perceptions). Internal consistency for all mPNES scores was deemed adequate across a large representative sample of older adults, i.e., Cronbach’s alphas, ⍺ ≥ 0.74 with the exception of a lower Cronbach’s ⍺ for the ambient environment score, ⍺ = 0.57 [36].

### 2.3. Census Tract Social Vulnerability

Participants provide their address at study entry given that study visits are traditionally conducted in a participant’s home. Prior to each subsequent annual study visit, addresses are reviewed with participants and changes documented. At the time of geocoding, addresses were reviewed and clerical errors corrected prior to using internal Geographic Information Systems (GIS) mapping via ESRI ArchMap (v10.8) and then ArcGIS Pro (v 3.6.2) after the ESRI upgrade in 2024 as well as US Census TigerLine data [6]. Geocoded baseline address data were used for the current study to derive Census tract determinations with the 2000 Census covering study visit years 1997 through 2009 and the 2010 Census covering study visit years 2010 through 2019. It is important to note that 172 of our 224 Latino participants (i.e., 77%) never moved from their geocoded baseline address during the years included in this analysis (i.e., 1997 through 2019). Given the degree of prospective residential stability, we felt reasonably confident in assuming a similar degree of retrospective residential stability (to suggest retrospective as well as prospective neighborhood-level exposure) despite not having address information for participants prior to study entry.

Described in detail elsewhere [6], the CDC Social Vulnerability Index (SVI) through 2019 [4,37] ranks US Census tracts based on 15 community-level factors categorized into (1) socioeconomic status, (2) household characteristics, (3) minority status and language, and (4) housing and transportation. Given its measurement of multiple indices associated with non-medical factors known to negatively impact health and health differences between populations [38,39,40], the SVI is increasingly being applied in cognitive and brain health research [6,8]. Participants’ analytic baseline year was used to determine the SVI year of data used for score determination (e.g., 2018 and 2019 analytic baseline years employed 2018 SVI data). SVI scores range from 0 to 1 with higher scores indicating higher neighborhood-levels of social vulnerability.

Given the timing of project funding, there was a temporal lag between SVI determination and administration of the mPNES. To determine whether the geocoded baseline visit (i.e., the earlier time point for most participants) could be used to maximize longitudinal cognitive follow-up, we assessed the correlation between these neighborhood measurements with and without adjusting for this time lag. Unadjusted Pearson Produce Moment Correlations of neighborhood health indicated a negative correlation, r(224) = −0.37, *p* < 0.0001, which was unchanged after adjusting for the time from geocoded baseline to the first mPNES administration, r(224) = −0.37, *p* < 0.0001. Accordingly, we used mPNES scores alongside geocoded baseline data to maximize longitudinal cognitive follow-up for analyses described below.

### 2.4. Cognition

Cognitive tests assessed five domains and were administered at annual study visits. Individual test scores were combined to represent episodic memory (two immediate and delayed story recall tests; word list learning, delayed memory, and recognition), semantic memory (confrontation naming; word reading; verbal fluency), working memory (digits forward and backward; digit ordering), perceptual speed (Stroop subtests; symbol digit modality; number comparisons), and visuospatial ability (line orientation; progressive matrices). Raw scores were converted to z-scores using the baseline mean and standard deviation (SD) of the entire cohort. They were then averaged for each of the five cognitive domains. A global cognitive function score was also derived by averaging a person’s standard scores across all test scores. Psychometric information on these summary scores has been deemed adequate [33,41].

### 2.5. Motor Gait Covariate

Motor gait is not only associated with cognitive and brain health [42], it also impacts one’s ability to engage with the physical and social environment [43]. Thus, we included a composite measure of this lower extremity function as a secondary covariate in analyses. Motor gait combined metrics of walking and turning 360 degrees. Specifically, participants walked a distance of 8 feet twice and turned 360 degrees twice. Time in seconds and number of steps required to walk the distance and to turn were recorded and reciprocated (1/‘original value’) so that larger values indicated less time and fewer steps. The four resulting values were each divided by the relevant mean of the entire cohort and then averaged to yield a composite measure of motor gait [44].

### 2.6. Statistical Analysis

Descriptive summaries of all variables were conducted. Additionally, analytic and graphic examinations of baseline data confirmed model assumptions were adequately met. Linear mixed effects regression models assessed the relationship of perceptions of neighborhood health (mPNES) with the level of and change in cognitive functioning (global and domain-specific cognition, separately). Additional terms in all models included age, sex, and education on both the intercept and the slope (i.e., interactions of each variable with time in years in the study) as well as a time-varying covariate representing cognitive administration (i.e., home visit vs. telephone). Within-participant correlation was captured by random intercepts and slopes. To investigate whether mPNES modified previously null associations between neighborhood social vulnerability (SVI) and cognition, we re-ran linear mixed effects models outlined above adding additional terms for SVI, and all relevant two-way (i.e., SVI × time in study, SVI × mPNES) and three-way (SVI × mPNES × time) interactions. We then conducted a similar series of secondary analyses using the three PCA-derived mPNES composites scores, i.e., community, health, and esthetic, as related to our singular metric of global cognition. We focused on global cognition only given the multiple comparison issues of considering three domain-specific perceptions and five cognitive domains. All primary and secondary linear regression models were further adjusted for motor gait. Lastly, and for primary models only, sensitivity analyses were conducted excluding individuals who relocated from their baseline address. All analyses were conducted using SAS/STAT software version 9.4 (SAS Institute Inc., Cary, NC, USA). Statistical significance was set at *p* < 0.05, two-sided. All data used in this manuscript are available upon request at www.radc.rush.edu.

## 3. Results

### 3.1. Participant Characteristics

**Figure 1:**
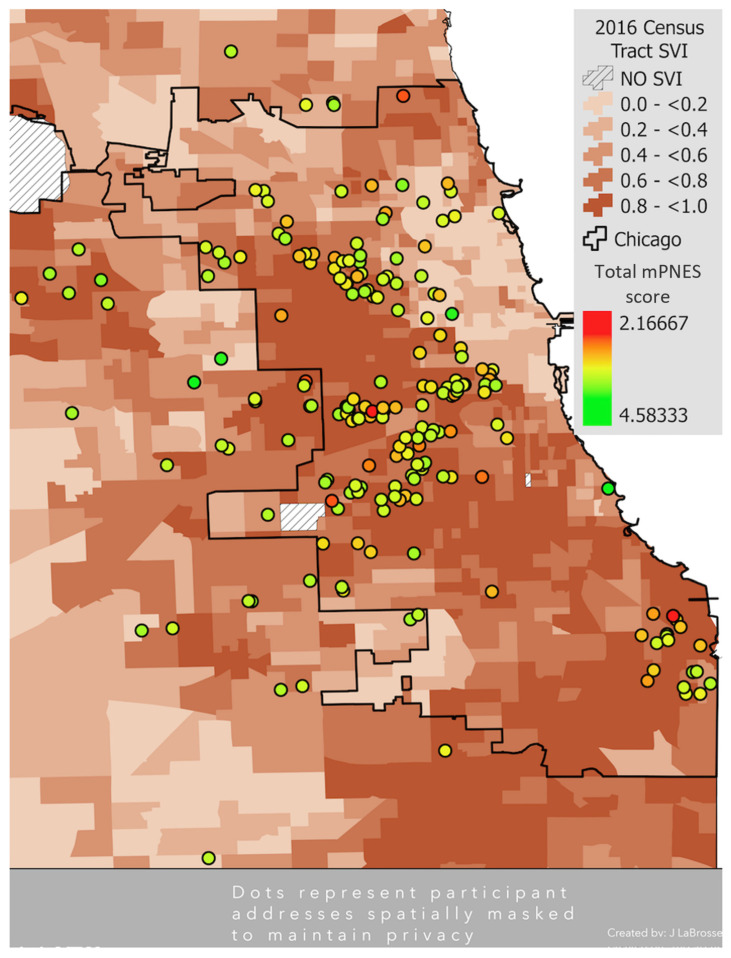
Latino participant locations and individual-level neighborhood perceptions (red-to-green = lower-to-higher total modified Perceptions of Neighborhood Environment Scale; mPNES) overlaid on representative (2016) Census-level Social Vulnerability Index scores (SVI; darker colors = greater vulnerability).

Participants were, on average, 70 years of age, predominantly female (~79%), and reported approximately 11 years of education. Participants reported a relatively neutral opinion of their neighborhoods (neither agree nor disagree) except for their tendency to disagree with positive statements regarding their neighborhood’s health opportunities. The average SVI score was 0.67, suggesting that, overall, participants lived in areas of Chicago (i.e., Census tracts) that are 67% more socially vulnerable than other Census tracts. Table 1 reports additional participant characteristics. Figure 1 displays participants’ mPNES scores overlaid on their neighborhood SVI scores.

### 3.2. mPNES and Cognition

Adjusted linear mixed effects models resulted in a significant association between mPNES scores and level of but not change in global cognition. Specifically, more positive overall perceptions were associated with higher baseline global cognitive functioning only (Table 2). This appeared to be driven by a more positive sense of community cohesiveness—the only sub-score associated with baseline global cognition (estimate = 0.135, standard error, SE = 0.055, *p* = 0.014). More positive perceptions of neighborhood-level health opportunities were associated with slower rates of decline in global cognition (estimate = 0.010, SE = 0.005, *p* = 0.044). For more details on all mPNES sub-scores see Table S1. Estimates of the association between mPNES total score and baseline global cognition did not change with the addition of motor gait (Table S2) although the *p*-value no longer met the threshold for significance (estimate = 0.054, SE = 0.029, *p* = 0.062); mPNES sub-scale results remained the same (Table S1). Perceptions of the ambient environment were not associated with level or change in global cognition (*p* ≥ 0.18), regardless of gait (Table S1).

Total mPNES scores were positively associated with working memory but at baseline only; baseline associations were also seen between total mPNES and visuospatial ability (*p*-values ≤ 0.049; see Table 2 for model details). Only the association between total mPNES and working memory remained significant after additional adjustment for gait (estimate = 0.100, SE = 0.044, *p* = 0.023). mPNES was not associated with any other cognitive domain at baseline or with changes in domain-specific cognition (Table 2), regardless of gait (Table S2).

### 3.3. Effect Modification

**Figure 2:**
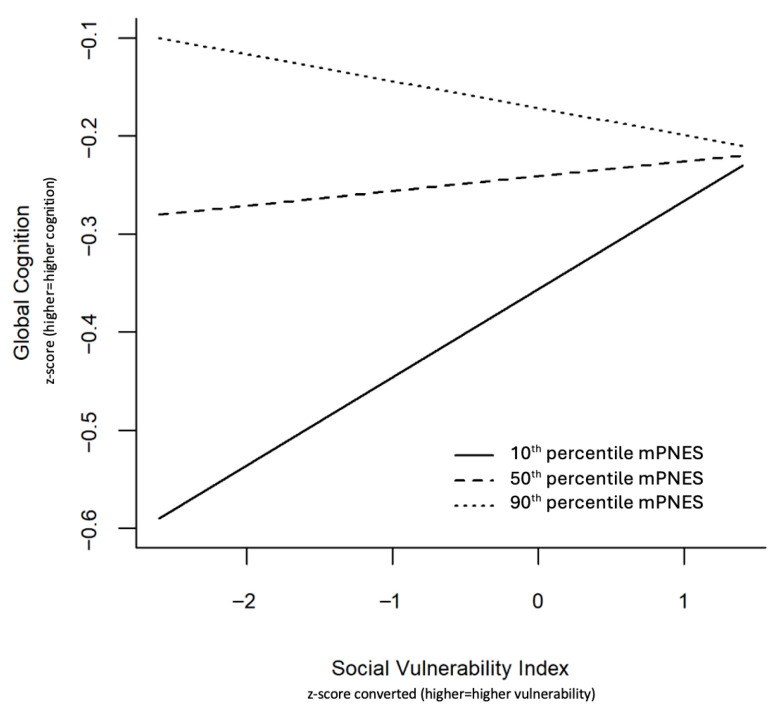
Results of the effect modification of the modified Perception of Neighborhood Health Scale (mPNES) on the relationship between the Census-defined Social Vulnerability Index (SVI) and global cognition z-score composite at analytic baseline adjusting for relevant confounders (chosen percentiles are purely for ease of visualization).

When mPNES and SVI were considered within the same demographically adjusted linear mixed effects models (Table 3), their two-way interaction was associated with levels of global cognitive functioning (estimate = −0.055, SE = 0.029, *p* = 0.063). Only after additional adjustment for gait did this association reach the threshold for significance (estimate = −0.060, SE = 0.029, *p* = 0.041). As shown in Figure 2, higher individual-level neighborhood perceptions modified the association of lower Census-level vulnerability and higher global cognition, but only for individuals living in objectively less vulnerable neighborhoods. Specifically, for individuals living in the most vulnerable neighborhoods, positive neighborhood perceptions do not exert any buffering effect on cognition. The mPNES total score continued to show a positive association with level of but not change in global cognitive function (Table 3), regardless of gait.

When considering mPNES sub-scores as separate predictor variables instead of total mPNES (Table S3), the community cohesiveness sub-score modified the relationship between SVI and global cognition at baseline. Specifically, the level estimate of the interaction between community sub-scores and SVI was significant (estimate = −0.120, SE = 0.055, *p* = 0.031) and the level main effect of SVI on global cognition was also significant (estimate = 0.466, SE = 0.209, *p* = 0.027). More generally, the community cohesiveness sub-score continued to show a positive association with levels of global cognition (estimate = 0.156, SE = 0.058, *p* = 0.007). None of the community cohesiveness sub-score results changed after additional adjustments for gait (Table S3). No effect modification of health or ambient perceptions on relationships between SVI and global cognition were noted (Table S3).

No two- or three-way interactions of total mPNES and SVI were significant when considering level of or change in domain-specific cognitive functions (Table 3); however, mPNES total scores modified the main effect of SVI on episodic memory regardless of gait. Specifically, higher SVI scores were associated with higher episodic memory scores at baseline (Table 3), an association not reported in our prior publication. Positive associations between total mPNES and baseline levels of working memory continued to remain significant regardless of adjustments for gait. No other associations of interest reached the threshold for significance (Table 3).

### 3.4. Sensitivity Analyses

As noted above, 52 participants (23% of the overall analytic sample) reported at least one address change during their cohort study participation. This left 172 participants contributing to our sensitivity analyses; given this relatively small sample size, these analyses should be considered exploratory secondary to a potential lack of power.

Excluding participants who relocated did not alter initially reported estimates between total mPNES scores and cognition, regardless of gait; the exclusion did, however, affect *p*-levels (*p*-values ≥ 0.057). In fact, the only association that approached the threshold for significance after excluding participants who relocated was between total mPNES scores and levels of working memory, and only after additional adjustments for gait (estimate = 0.095, SE = 0.050, *p* = 0.057). A similar pattern of relatively unchanged estimates but above-threshold *p*-values after excluding participants who relocated was seen when investigating effect modification of total mPNES scores on associations between SVI and cognition. There was one exception: the two-way interaction of SVI by time was affected by the inclusion of mPNES in the model, suggesting that higher SVI scores may be associated with faster declines in visuospatial ability for those who did not relocate from their baseline address (estimate = −0.014, SE = 0.006, *p* = 0.019), and the three-way interaction of mPNES, SVI, and time approached significance (estimate = −0.012, SE = 0.006, *p* = 0.055). These results remained relatively stable with the further adjustment for gait (two-way estimate = −0.013, SE = 0.006, *p* = 0.036; three-way estimate = −0.011, SE = 0.006, *p* = 0.065).

## 4. Discussion

In a sample of 224 older Latinos, we tested associations between individual-level perceptions of neighborhood health and cognitive functioning and the moderating role of these perceptions on previously documented null relationships between Census-level neighborhood vulnerability and cognition [6]. More positive neighborhood perceptions were associated with higher levels of global cognition and working memory, regardless of adjustments. When considered jointly, individual-level neighborhood perceptions modified associations between Census-level neighborhood health and cognition but only for older Latinos living in less vulnerable neighborhoods. Overall, results demonstrate the utility of incorporating individual-level experiential dimensions of neighborhood context when investigating associations of broader Census-level neighborhood conditions with cognition in older Latino adults. Results do not, however, suggest a potential ‘blanket’ buffering role of these individual-level experiences on cognition, especially once neighborhood-level vulnerability exceeds a certain threshold.

This work contributes to the literature assessing neighborhood health and cognitive outcomes in several ways. First, to our knowledge, this study is one of the first to investigate Census- and individual-level metrics of neighborhood health in relation to cognitive outcomes generally, and the first within older Latino adults specifically. Other studies have reported separate contributions for each of these neighborhood health measures on cognition [45]; however, few investigate their interplay [15]. Thus, we highlight the importance of considering multiple dimensions of neighborhood environment when investigating cognitive aging in Latinos. Second, this study extends previous work documenting associations focused exclusively on neighborhood perceptions and cognition conducted in predominantly non-Latino populations [15,45,46] by adding to the two other US-based studies of Latinos [16,17] demonstrating the relationship between neighborhood perceptions and cognition in this high-risk population. Third, our inclusion of domain-specific cognitive functioning provides greater context to previous neighborhood perception studies focused on global cognition [45,46,47] and confirm prior results involving US-based Latinos employing similar tests of working memory [16,17]. Fourth, by incorporating Census- and individual-level measures of neighborhood characteristics in our work, we provide empirical support for prior assertions—both experimental [15,23,24] and theoretical [19,20]—that perceptions of neighborhood health add important community-based perspectives value to Census-driven neighborhood studies, particularly in high-risk populations [21,22].

Both overall perceptions and domain-specific perceptions of community cohesiveness modified relationships between Census-level neighborhood vulnerability and global as well as domain-specific (episodic memory) cognition not previously seen in our cohort [6]. These effects were seen for baseline levels of (as opposed to longitudinal changes in) cognition and were often contrary to expectations (discussed in more detail below). Further, while the ability of neighborhood perceptions to modify associations between lower levels of neighborhood vulnerability and higher levels of global cognition was notable, it was restricted to individuals living in less vulnerable neighborhoods. In fact, when considering the totality of this result, neighborhood perceptions made the relationship between neighborhood vulnerability and levels of global cognition more negative. This finding only reached the threshold for significance when additionally adjusting for a proxy for mobility. This suggests that one’s ability to ambulate within the environment changes the dynamics of how perceptions impact associations between that environment and cognition. It may also suggest that individual-level perceptions confer an increased risk of adverse outcomes when paired with Census-level measures [14,15]. More work is needed to fully understand these relationships.

When investigating Latinos’ perceptions of their neighborhood and cognition, regardless of whether neighborhood vulnerability was also considered, more positive overall perceptions, as well as more positive community cohesiveness and health opportunities, were positively associated with level of more so than change in cognition. Findings for overall (i.e., total) perceptions and global cognition are in keeping with US-based studies of non-Latino [15,45,46] and Latino [16] cohorts as well as a study in Brazil [48]. Prior studies of domain-specific perceptions in other diverse populations also support our results of positive relationships between perceived community cohesion and health opportunities with level of as well as change in cognition, respectively [16,28,47,48]. Taken together, these findings reinforce socio-environmental models [19,20] that suggest nuanced layers—including resident-based individual-level information—contribute to the study of health and aging.

There are several potential pathways through which participants’ perceptions of their neighborhood may be associated with cognition generally and may shape associations between measures of neighborhood vulnerability and cognition more specifically. Generally, neighborhood perceptions are thought to more accurately reflect how an individual interacts with their environment and engages in health-related behaviors known to promote successful cognitive and brain aging [45,46] (e.g., walking, [49,50]). Furthermore, there is a known protective effect of more positive perceptions of neighborhood collective efficacy (i.e., community) on biological aging [51]. Consistent with this, the only significant longitudinal result was the association between more positive perceptions of neighborhood-level health opportunities and slower declines in global cognition. This does not, however, explain contradictory findings (e.g., higher Census-derived neighborhood vulnerability associated with higher levels of episodic memory), and findings made more negative by accounting for perceptions underscoring the complexity of these relationships. Perhaps perceptions of neighborhood health that intimate a more positive outlook run afoul of neighborhood vulnerability at a certain threshold. Subsequently, a person may need to rely on past positive autobiographical memories of their neighborhood maintained by a robust episodic memory system to bolster positive perceptions in the face of negative realities. This explanation is tentative at best, particularly given the cross-sectional nature of most results preclude discussions of directionality.

Results of this study should be considered within the context of additional limitations. For example, our measure of neighborhood perceptions was given, on average, 5 years after geocoded baseline, and thus, 5 years after the determination of objective neighborhood health. While this time lag did not appear to affect the relationship between objective and subjective neighborhood health metrics, and a certain degree of lag allows time for our exposures to influence perceptions, the lag may nonetheless have allowed for changes in Census-tract characteristics. For example, based on American Community Survey data between 2009 and 2024—a range three times that of this study’s average time lag, yet encompassing the timeframe of our longitudinal studies—neighborhood-level shifts included downtown revitalization, gentrification in some formerly low-income areas, as well as persistent economic differences, rising housing costs, and high poverty in others. While Census-level changes do not always correspond to changes in neighborhood perceptions [12], they may impact a more mPNES-concurrent measure of SVI. Thus, validation work is needed using simultaneously acquired objective and subjective neighborhood measures as it relates to cognitive outcomes. Given several findings were sensitive to model adjustments (e.g., lost or gained significance after including gait) and sensitivity analyses, their interpretation should be considered within the limited robustness of their effects and the potential for Type I error inflation. The inclusion of only older Latinos from a single urban US location, justified based on our prior research [6], nonetheless limits generalizability. Furthermore, while we focused on the role of perceived neighborhood health as an effect modifier of the relationship between objective neighborhood health and cognition, the possibility remains that subjective perceptions may also be a mediator of this same relationship as reported in other, predominantly non-Latino White populations (e.g., [15]). Lastly, limitations inherent in our objective measure of neighborhood health are also limitations for this study.

## 5. Conclusions

This study demonstrates the importance of considering individual-level neighborhood perceptions both alone and in conjunction with Census-level measures of neighborhood health when examining relationships of these predictors with cognition in older Latinos. While these perceptions may unmask relationships between neighborhood vulnerability and cognition not previously noted, some findings were reliant on model specifications and, in some cases, only existed below a certain threshold of neighborhood-level vulnerability. This may suggest a more exploratory nature of some results. It may also suggest that other cultural and/or environmental factors present in Latino communities should be incorporated into future studies. Overall, it is our hope that this work will contribute to ongoing dialogue supporting broader socio-environmental inclusions in research and interventions focused on cognitive aging in older Latinos. For example, a broader focus for improvement projects in vulnerable areas that not only rebuilds neighborhood-level infrastructure, but also promotes environments that support individual-level engagement relevant to cognitive health.

## Figures and Tables

**Table 1:** Participant characteristics and key variables of interest.

	Latino Adults (*N* = 224)
Age (years)	70.35 (6.57)
Sex (female:male)	177:47
Education (years)	10.88 (4.92)
mPNES Total	3.46 (0.41)
Community Cohesiveness	3.68 (0.542)
Health Opportunities	2.12 (0.65)
Ambient Environment	3.26 (0.72)
Social Vulnerability Index	0.67 (0.24)
Participants who Relocated (ever; n, %)	52 (23.21%)
Global Cognition	−0.15 (0.53)
Episodic Memory	0.01 (0.56)
Semantic Memory	−0.10 (0.77)
Working Memory	−0.68 (0.79)
Visuospatial Ability	−0.24 (0.78)
Perceptual Speed	−0.03 (0.78)
Cognitive Visits	6.77 (2.50)

Note: Values are mean (standard deviation) unless otherwise noted mPNES = modified (12-item) Perception of Neighborhood Environment Scale.

**Table 2:** Results of adjusted linear mixed effects regression models on the relationship between mPNES and cognition.

	Global Cognition	Episodic Memory	Semantic Memory	Working Memory	Visuospatial Ability	Perceptual Speed
Age	−0.022 ± 0.004 *p* < 0.0001	−0.019 ± 0.005 *p* = 0.0003	−0.025 ± 0.006 *p* = 0.0001	−0.015 ± 0.007 *p* = 0.024	−0.014 ± 0.006 *p* = 0.026	−0.039 ± 0.006 *p* < 0.0001
Male Sex	−0.113 ± 0.070 *p* = 0.11	−0.165 ± 0.084 *p* = 0.051	−0.100 ± 0.102 *p* = 0.32	0.018 ± 0.106 *p* = 0.87	0.144 ± 0.099 *p* = 0.15	−0.221 ± 0.097 *p* = 0.024
Education	0.056 ± 0.006 *p* < 0.0001	0.039 ± 0.007 *p* < 0.0001	0.053 ± 0.009 *p* < 0.0001	0.072 ± 0.009 *p* < 0.0001	0.053 ± 0.008 *p* < 0.0001	0.082 ± 0.008 *p* < 0.0001
**mPNES**	**0.058 ± 0.029** *p* = 0.044 *	**0.014 ± 0.034** ***p* = 0.69**	**0.060 ± 0.042** ***p* = 0.16**	**0.096 ± 0.044** *p* = 0.030 *	**0.080 ± 0.040** *p* = 0.049 *	**0.057 ± 0.040** ***p* = 0.16**
Age × time	−0.001 ± 0.000 *p* = 0.11	−0.003 ± 0.001 *p* = 0.0002	−0.000 ± 0.001 *p* = 0.556	0.001 ± 0.001 *p* = 0.31	−0.002 ± 0.001 *p* = 0.0003	−0.001 ± 0.001 *p* = 0.15
Male Sex × time	−0.010 ± 0.008 *p* = 0.19	−0.017 ± 0.012 *p* = 0.16	−0.009 ± 0.012 *p* = 0.46	−0.013 ± 0.011 *p* = 0.24	−0.009 ± 0.012 *p* = 0.44	−0.001 ± 0.011 *p* = 0.91
Education × time	−0.000 ± 0.001 *p* = 0.96	0.001 ± 0.001 *p* = 0.41	−0.001 ± 0.001 *p* = 0.46	0.001 ± 0.001 *p* = 0.35	0.002 ± 0.001 *p* = 0.080	−0.003 ± 0.001 *p* = 0.0007
**mPNES** × **time**	**0.003 ± 0.003** ***p* = 0.37**	**0.005 ± 0.005** ***p* = 0.33**	**0.006 ± 0.005** ***p* = 0.22**	**0.001 ± 0.005** ***p* = 0.75**	**−0.003 ± 0.005** ***p* = 0.50**	**0.002 ± 0.004** ***p* = 0.59**

NOTE: values represent standardized beta estimates ± standard error (*p*-value) with bolded entries representing our predictors of interest; * represents associations that met statistical significance of *p* < 0.05; mPNES = modified (12-item) Perception of Neighborhood Environment Scale; time = time in study. All models also contained a time-varying covariate representing cognitive administration (i.e., home visit versus telephone).

**Table 3:** Results of adjusted linear mixed effects regression models testing effect modification of mPNES on the relationship between SVI and cognition.

	Global Cognition	Episodic Memory	Semantic Memory	Working Memory	Visuospatial Ability	Perceptual Speed
**mPNES**	**0.065 ± 0.030** ***p* = 0.034**	0.038 ± 0.036 *p* = 0.29	0.066 ± 0.044 *p* = 0.14	**0.091 ± 0.046** ***p* = 0.050**	**0.090 ± 0.043** ***p* = 0.038**	0.047 ± 0.042 *p* = 0.27
**SVI**	0.019 ± 0.031 *p* = 0.55	**0.075 ± 0.037** ***p* = 0.047**	0.017 ± 0.046 *p* = 0.72	−0.018 ± 0.048 *p* = 0.70	0.031 ± 0.044 *p* = 0.49	−0.029 ± 0.044 *p* = 0.50
**mPNES** × **SVI**	−0.055 ± 0.029 *p* = 0.063	−0.036 ± 0.035 *p* = 0.29	−0.068 ± 0.043 *p* = 0.11	−0.074 ± 0.044 *p* = 0.098	−0.061 ± 0.041 *p* = 0.14	−0.047 ± 0.040 *p* = 0.25
**mPNES** × **time**	0.003 ± 0.003 *p* = 0.43	0.004 ± 0.005 *p* = 0.43	0.007 ± 0.005 *p* = 0.16	0.001 ± 0.005 *p* = 0.85	−0.006 ± 0.005 *p* = 0.25	0.004 ± 0.005 *p* = 0.40
**SVI** × **time**	−0.000 ± 0.003 *p* = 0.92	−0.002 ± 0.005 *p* = 0.73	0.004 ± 0.005 *p* = 0.43	−0.001 ± 0.005 *p* = 0.79	−0.007 ± 0.005 *p* = 0.15	0.005 ± 0.005 *p* = 0.31
**mPNES** × **SVI** × **time**	0.001 ± 0.003 *p* = 0.76	0.000 ± 0.005 *p* = 0.94	0.006 ± 0.005 *p* = 0.24	0.002 ± 0.005 *p* = 0.73	−0.007 ± 0.005 *p* = 0.15	0.001 ± 0.005 *p* = 0.81

NOTE: values represent standardized beta estimates ± standard error (*p*-value) for our predictors of interest as well as effect modifier terms only for ease of comparison although models also contained terms for age, sex, education, the interaction of these covariates with time (in study) and a time-varying covariate representing cognitive administration (i.e., home visit versus telephone); SVI = Social Vulnerability Index, mPNES = modified (12-item) Perception of Neighborhood Environment Scale. Bolded entries met statistical significance *p* < 0.05.

## Data Availability

All data used in this manuscript are available upon request at www.radc.rush.edu.

## Supplementary Materials

The following supporting information can be downloaded at: https://www.mdpi.com/article/10.3390/ijerph23060714/s1, Table S1: Results of adjusted linear mixed effects regression models on the relationship between subjective neighborhood health sub-scores and cognition; Table S2: Results of adjusted linear mixed effects regression models on the relationship between subjective neighborhood health metrics and cognition with additional adjustment for gait; Table S3: Results of adjusted linear mixed effects regression models testing the effect modification of subjective neighborhood health sub-scores on the relationship between objective neighborhood health and cognition.

## Abbreviations

The following abbreviations are used in this manuscript:

mPNES	modified Perception of Neighborhood Environment Scale
SVI	Social Vulnerability Index
CDC	Centers for Disease Control
AD	Alzheimer’s disease
ADRD	AD-related dementias
RADC	Rush Alzheimer’s Disease Center
MAP	Rush Memory and Aging Project
MARS	Minority Aging Research Study
GIS	Geographic Information Systems
SD	standard deviation
SE	standard error
