# Supplementary material for: The Modifying Effect of Individual-Level Neighborhood Perceptions on the Relationship Between Census-Level Neighborhood Social Vulnerability and Cognition in Older Latinos
Source: Int J Environ Res Public Health. 2026 May 27;23(6):714. doi: 10.3390/ijerph23060714 (PMC13300457; doi:10.3390/ijerph23060714)
Supplement: Supplementary file 1 [file ijerph-23-00714-s001.zip › ijerph-4250822-supplementary.pdf]

Table S1. Results of adjusted linear mixed effects regression models on the relationship between subjective neighborhood health sub-scores and cognition

	**Global Cognition**	**Global Cognition** *additionally adjusted for gait*
**Community**	**0.135±0.055 p=0.014**	0.127±0.055 p=0.021
**Community*time**	-0.001±0.006 p=0.84	ns
**Health**	0.009±0.046 p=0.84	ns
**Health*time**	**0.010±0.005 p=0.044**	0.011±0.005 p=0.014
**Ambient**	0.005±0.040 p=0.89	ns
**Ambient*time**	0.006±0.005 p=0.18	ns

NOTE: values represent standardized beta estimates ± standard error (p-value) for our predictors of interest with bolded entries representing associations meeting threshold for statistical significance of $p<0.05$. Models contained terms for age, sex, education, the interaction of these covariates with time (in study) and a time-varying covariate representing cognitive administration (i.e., home visit versus telephone).

Table S2. Results of adjusted linear mixed effects regression models on the relationship between subjective neighborhood health metrics and cognition with additional adjustment for gait

	**Global Cognition**	**Episodic Memory**	**Semantic Memory**	**Working Memory**	**Visuospatial Ability**	**Perceptual Speed**
Age	-0.020±0.004 p<0.0001	-0.016±0.005 p=0.003	-0.024±0.007 p=0.0003	-0.014±0.007 p=0.044	-0.012±0.006 p=0.064	-0.037±0.006 p<0.0001
Male Sex	-0.114±0.070 p=0.107	-0.168±0.085 p=0.050	-0.101±0.103 p=0.33	0.027±0.106 p=0.80	0.137±0.099 p=0.17	-0.214±0.095 p=0.026
Education	0.055±0.006 p<0.0001	0.037±0.007 p<0.0001	0.054±0.009 p<0.0001	0.071±0.009 p<0.0001	0.052±0.008 p<0.0001	0.080±0.008 p<0.0001
**mPNES**	**0.054±0.029 p=0.062**	**0.015±0.035 p=0.66**	**0.058±0.042 p=0.17**	**0.100±0.044 p=0.023***	**0.071±0.041 p=0.083**	**0.050±0.039 p=0.206**
Age*time	-0.001±0.000 p=0.076	-0.003±0.001 p=0.0001	-0.000±0.001 p=0.65	0.000±0.001 p=0.53	-0.002±0.001 p=0.0006	-0.001±0.001 p=0.20
Male Sex*time	-0.009±0.008 p=0.26	-0.016±0.012 p=0.19	-0.007±0.012 p=0.54	-0.013±0.011 p=0.27	-0.008±0.012 p=0.51	-0.001±0.011 p=0.96
Education*time	-0.000±0.001 p=0.87	0.001±0.001 p=0.53	-0.001±0.001 p=0.35	0.001±0.001 p=0.30	0.002±0.001 p=0.072	-0.003±0.001 p=0.0007
**mPNES*time**	**0.004±0.003 p=0.25**	**0.004±0.005 p=0.38**	**0.007±0.005 p=0.18**	**0.001±0.005 p=0.78**	**-0.002±0.005 p=0.69**	**0.003±0.005 p=0.55**

NOTE: values represent standardized beta estimates±standard error (p-value) with bolded entries representing our predictors of interest and * represents associations that met statistical significance of p<0.05; mPNES=modified (12-item) Perception of Neighborhood Environment Scale, time=time in study. All models also contained a time-varying covariate representing cognitive administration (i.e., home visit versus telephone).

Table S3. Results of adjusted linear mixed effects regression models testing the effect modification of subjective neighborhood health sub-scores on the relationship between objective neighborhood health and cognition

	**Global Cognition**	**Global Cognition** *additionally adjusted for gait*
Community	**0.156±0.058 p=0.007**	0.150 (0.058,0.0102)
Community*time	-0.002±0.007 p=0.74	ns
SVI	**0.466±0.209 p=0.027**	0.486 (0.208,0.0204)
SVI*time	-0.012±0.023 p=0.62	ns
Community*SVI	**-0.120±0.055 p=0.031**	-0.125 (0.055,0.0240)
Community*SVI*time	0.003±0.006 p=0.67	ns
Health	0.007±0.046 p=0.88	ns
Health*time	**0.010±0.005 p=0.048**	0.011 (0.005,0.0199)
SVI	0.066±0.097 p=0.49	ns
SVI*time	0.004±0.010 p=0.70	ns
Health*SVI	-0.034±0.043 p=0.42	ns
Health*SVI*time	-0.002±0.005 p=0.63	ns
Ambient	0.004±0.041 p=0.93	ns
Ambient*time	0.006±0.005 p=0.18	ns
SVI	-0.011±0.122 p=0.93	ns
SVI*time	-0.006±0.014 p=0.63	ns
Ambient*SVI	0.001±0.036 p=0.98	ns
Ambient*SVI*time	0.002±0.004 p=0.64	ns

NOTE: values represent standardized beta estimates ± standard error (p-value) for our predictors of interest as well as effect modifier terms only for ease of comparison with bolded entries representing associations meeting threshold for statistical significance of $p<0.05$. Models also contained terms for age, sex, education, the interaction of these covariates with time (in study) and a time-varying covariate representing cognitive administration (i.e., home visit versus telephone); SVI=Social Vulnerability Index. Results did not appreciably change with further adjustment for gait as noted; ns=not significant
